# Mucous Cyst of the Clitoris: A Report of a Rare Case

**DOI:** 10.7759/cureus.113908

**Published:** 2026-08-03

**Authors:** Satoshi Akaishi, Hiroaki Kuwahara

**Affiliations:** 1 Plastic Surgery, Nippon Medical School Musashi Kosugi Hospital, Kanagawa, JPN

**Keywords:** clitoris, clitoroplasty, mucous cyst, urogenital sinus, vulvar cyst

## Abstract

Mucous cysts of the vulva most commonly arise from the vaginal vestibule and the inner surface of the labia minora, and to our knowledge, only a single previous case arising within the clitoris has been described.

We report the case of a 50-year-old multiparous woman who presented with a two-year history of asymptomatic progressive enlargement of the clitoris to 21 mm × 15 mm × 15 mm. Surgical excision under general anesthesia revealed a well-demarcated cyst containing yellow-white viscous mucoid fluid within the clitoral tissue. A compressed but morphologically intact clitoral glans was preserved, and clitoroplasty was completed using absorbable sutures. Histopathological examination revealed a unilocular cyst lined with a single layer of tall columnar epithelial cells with basally oriented nuclei and intraluminal amorphous mucoid material consistent with a mucous cyst. Differential diagnoses, including epidermal cysts, Bartholin duct cysts, mesothelial cysts, Gartner’s duct cysts, and Skene’s duct cysts, were excluded on clinical and histopathological grounds. No recurrence was observed at the one-year follow-up.

To our knowledge, this represents the second documented mucous cyst arising within the clitoris. Complete surgical excision with preservation of the clitoral glans was accomplished with satisfactory cosmetic results.

## Introduction

Cystic lesions of the vulva are uncommon. They include both embryologic (Bartholin, Skene’s, and Gartner’s duct cysts, mesothelial cysts of the canal of Nuck, and mucous cysts) and non-embryologic (epidermal inclusions and endometriotic cysts) entities [1-3]. Mucous cysts, the least frequently reported subtype, arise from ectopic urogenital sinus-derived mucous gland tissue and are found principally in the vestibule and on the medial labia minora [1,3]. The clitoris, an embryologically distinct structure [4], is only rarely affected by cystic lesions; the clitoral cysts reported to date have most often been epidermoid (squamous) cysts, which are a histologically distinct entity [5-7], and periclitoral cysts of other types remain uncommon [8]. Mucous cysts arising within the clitoris are exceptionally rare: to our knowledge, only a single prior case has been documented, a giant mesonephric (mucous) cyst reported by Bhuria et al. [9].

We report the case of a 50-year-old woman with a mucous cyst originating in the clitoral glans, which is, to our knowledge, the second such case to be described.

## Case presentation

**Figure 1:**
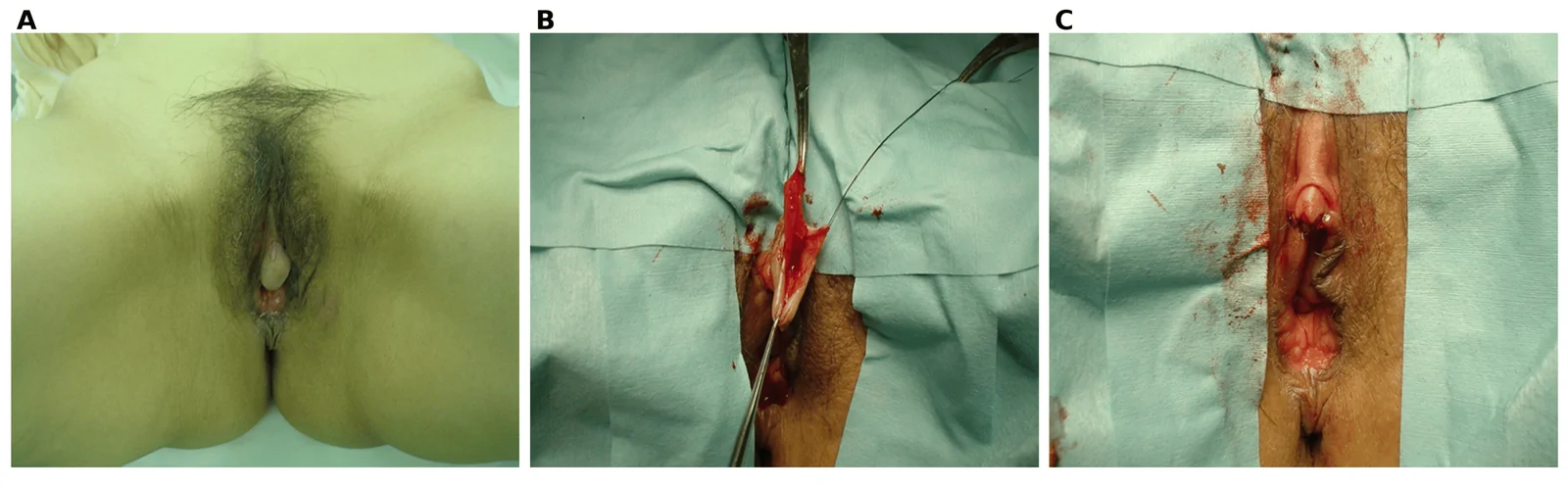
Clinical photographs (A) Preoperative appearance: the clitoris is diffusely and peduncularly enlarged to approximately 21 × 15 × 15 mm. The overlying skin is smooth and intact without erythema or ulceration. (B) Intraoperative finding: a well-demarcated cyst with clearly defined margins was identified within the clitoral substance and excised by sharp dissection. (C) Postoperative appearance: following excision of redundant skin and clitoroplasty with absorbable sutures, normal clitoral dimensions were restored with a satisfactory cosmetic result.

A 50-year-old multiparous woman was referred for progressive painless clitoral enlargement for two years without trauma, infection, hormonal therapy, or prior genital surgery. Examination revealed that the clitoris was diffusely and peduncularly enlarged (21 × 15 × 15 mm), with a soft, fluctuant distal portion and smooth, intact overlying skin (Figure 1A). There were no signs of virilization, such as hirsutism or voice change, and she had no history of androgen or anabolic steroid use. High-resolution ultrasonography demonstrated a well-circumscribed cystic lesion confined to the clitoris, without solid components or internal vascularity, excluding a solid neoplasm or a vascular lesion. Magnetic resonance imaging was not obtained, as the lesion was superficial, well circumscribed, and adequately characterized by ultrasonography. Diagnostic needle aspiration performed three days earlier was not informative.

Under general anesthesia, an incision at the tip of the mass revealed a yellow-white viscous mucoid fluid (Figure 1B). A well-demarcated cyst within clitoral tissue was excised in its entirety via sharp dissection, revealing a compressed but morphologically intact clitoral glans deep within the cyst cavity. The cyst separated from the surrounding tissue along a clean plane without adhesions and was removed intact, so that dissection within the clitoral body and identification of the dorsal neurovascular bundle were not required; the glans and its neurovascular supply were thereby left undisturbed. Redundant skin was excised, and clitoroplasty was completed using 5-0 polyglactin 910 for the deep layer and 6-0 nylon for the skin, restoring normal clitoral dimensions (Figure 1C). The postoperative course was uneventful. At her one-year follow-up, there was no recurrence, clitoral sensation was subjectively normal, and the patient was satisfied with the cosmetic outcome. She was not sexually active, so sexual function could not be formally assessed. Follow-up photography was declined given the sensitive anatomical site.

**Figure 2:**
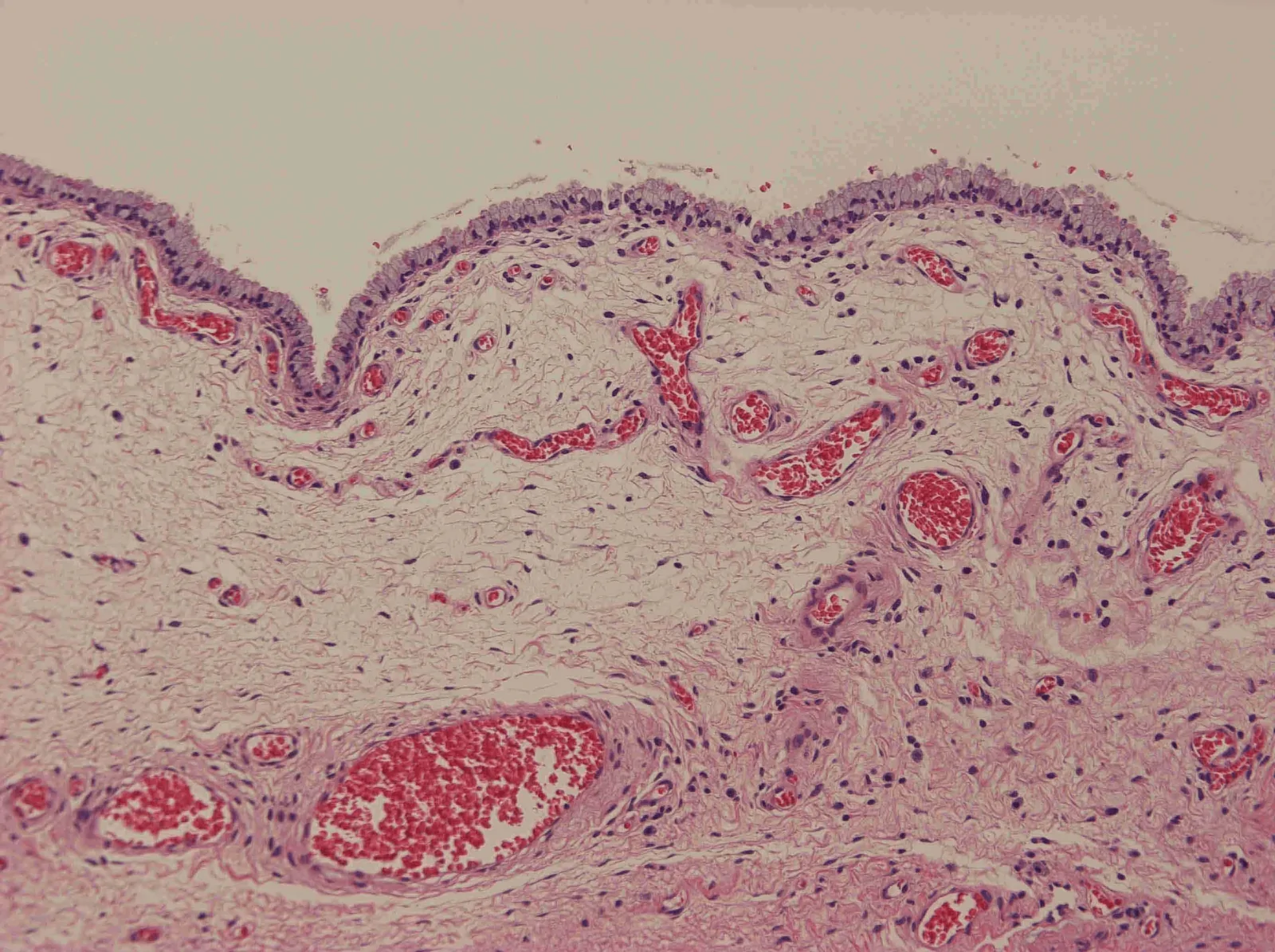
Histopathological examination Histopathology (hematoxylin and eosin, ×50) showing a unilocular cyst encapsulated by a single layer of tall columnar epithelium, with amorphous mucoid material within the cyst lumen, consistent with a mucous cyst.

Histopathology (hematoxylin and eosin staining) revealed a unilocular cyst lined with a single layer of tall columnar epithelial cells with basally polarized nuclei and amorphous mucoid luminal content (Figure 2) and no squamous epithelium, granular cell layer, ciliated cells, or smooth muscle. Bacteriological culture results were negative.

## Discussion

Histopathological features, including a unilocular cyst lined by tall mucin-secreting columnar epithelium with basally oriented nuclei, are defining criteria for a mucous cyst established by Robboy et al. [1], who identified small mucinous glands in 53% of autopsy vulvas and proposed obstruction of these glands as the cyst formation mechanism. This pattern has been confirmed in a subsequent clinical report [10].

Differential diagnoses were excluded on histopathological and anatomical grounds. Epidermal inclusion cysts show keratinizing stratified squamous epithelium with an intact granular layer and laminated keratin [3], inconsistent with the single-layer columnar lining observed. Bartholin duct cysts arise in the posterior vestibule, anatomically remote from the clitoris [3,10]. Mesothelial cysts of the canal of Nuck are lined by mesothelial cells rather than tall columnar epithelium [10]. Gartner’s duct cysts are lined by non-mucinous cuboidal to low columnar epithelium [10], and Skene’s duct cysts arise adjacent to the urethral meatus rather than the clitoris [3,10]. Immunohistochemistry was not performed. The diagnosis rested on morphology alone: a unilocular cyst lined by a single layer of tall, mucin-secreting columnar epithelium with basally oriented nuclei fulfils the histological criteria for a vulvar mucous cyst defined by Robboy et al. [1], and each of the principal differential diagnoses is distinguished by a lining of clearly different morphology, as outlined above. Immunohistochemistry, for example, for CK7, CK20, CEA, or calretinin, would be informative in histologically ambiguous cases but was not considered necessary here.

**Figure 3:**
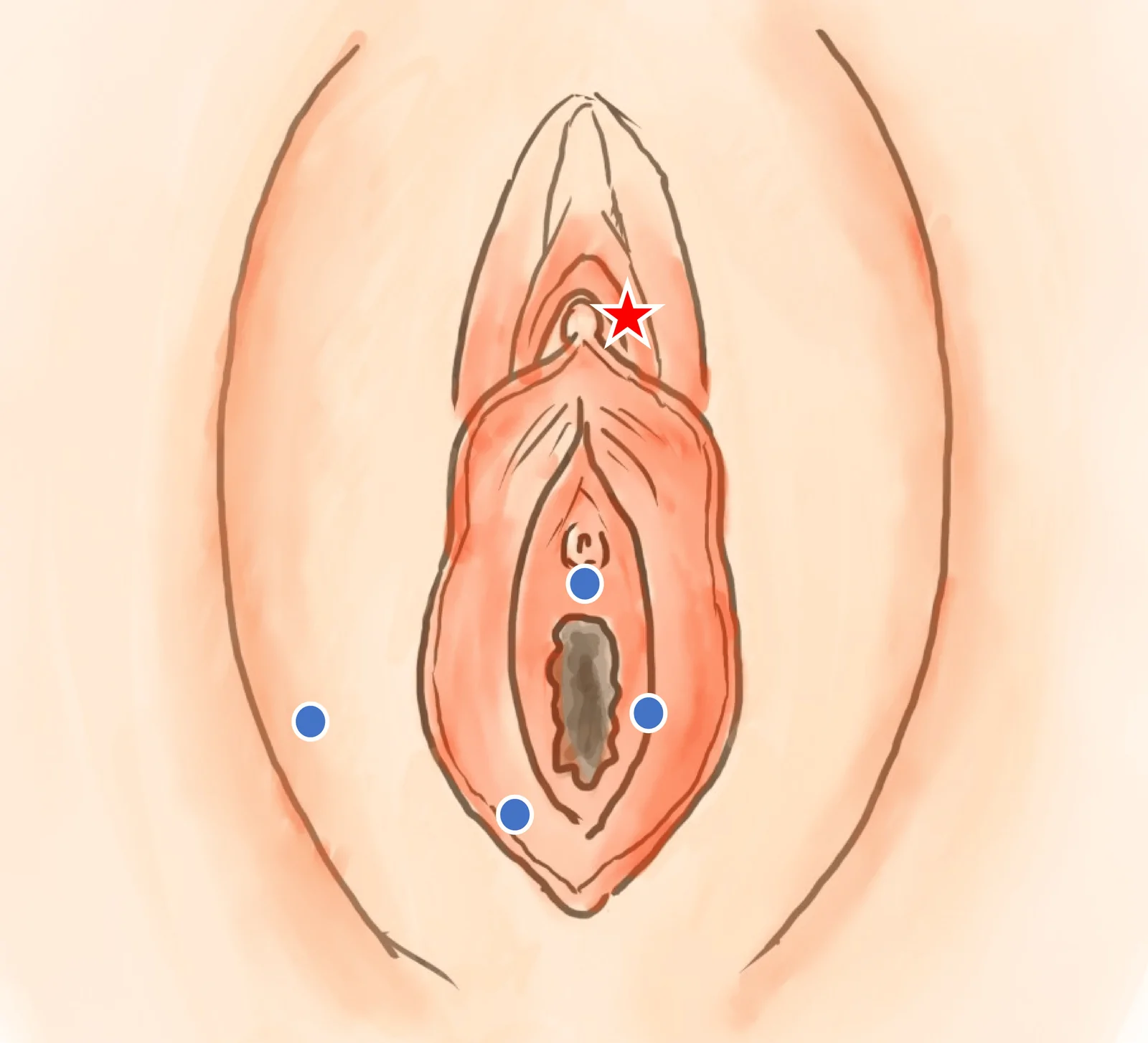
Anatomical distribution of vulvar mucous cysts Frontal view of the vulva. Blue circles indicate the anatomical zones in which vulvar mucous cysts have been reported: the vestibule and the medial labia minora, which are the typical sites [1,3], and the labia majora, the site of the few individually reported cases (Table 1) [10,11]. The red star indicates the present case, located within the clitoral glans. The base illustration was hand-drawn by the corresponding author, and the anatomical markers were overlaid by the authors using presentation software. No generative artificial intelligence was used to create this figure.

Mucous cysts arise from minor vestibular glands and are typically located in the vestibule and on the medial labia minora [1,3]; reported sizes range from 2 to 30 mm [3]. The few individually reported cases have involved the labia majora (Table 1) [10,11]. Cysts arising within the clitoris are exceptional: to our knowledge, the only prior report is the giant mesonephric (mucous) cyst described by Bhuria et al. [9], and the present case represents the second mucous cyst documented at this site (Figure 3 and Table 1). Whereas Bhuria et al. reported a mesonephric cyst, the present lesion was lined by urogenital sinus-type mucin-secreting columnar epithelium, indicating that clitoral mucous cysts of differing histogenesis may occur. We propose, as a hypothesis rather than a demonstrated mechanism, that the embryological basis of the present case lies in the shared urogenital sinus origin of the clitoral and vestibular mucosa [1]: ectopic mucous gland tissue, normally confined to the vestibule, may become incorporated within the developing clitoral glans and provide a substrate for cyst formation. A single case cannot establish this, and the mechanism remains speculative.

**Table 1:** Published individual case reports of vulvar mucous cysts * Year of surgery. Sizes are clinical measurements. Numbers in brackets refer to references.

Year	Author	n	Age (y)	Size (cm)	Location
2015	Bhuria et al. [9]	1	50	15 × 6	Clitoris
2025	Kumar et al. [10]	1	36	3 × 4	Left labia majora
2019	Campbell et al. [11]	1	29	2.5 × 4	Left labia majora
2026*	Present case	1	50	2.1 × 1.5	Clitoris

Clitoral enlargement itself has a broad differential diagnosis. It is most often hormonally mediated, reflecting androgen excess arising from congenital adrenal hyperplasia, polycystic ovary syndrome, androgen-secreting ovarian or adrenal tumors, or exogenous androgen use. Non-hormonal causes are rare and consist largely of mass lesions, including epidermoid inclusion cysts [5-7], periclitoral cysts of other types [8], and benign mesenchymal or vascular tumors. In the present patient, the enlargement was isolated, without any clinical feature of virilization or history of androgen exposure, and high-resolution ultrasonography demonstrated a purely cystic lesion; a hormonal cause was therefore considered excluded on clinical and imaging grounds, and endocrine assays were not performed. Ultrasonography has likewise proved decisive in a reported case of non-hormonal clitoromegaly caused by an epidermal inclusion cyst [12].

Surgery revealed that the cyst had expanded as a space-occupying lesion, compressing rather than replacing the clitoral glans. This insight supports a conservative approach in which complete excision preserves clitoral architecture with satisfactory cosmetic and functional results. Simple aspiration, as attempted at initial presentation, is not curative because the secretory lining remains intact. Complete surgical excision is the treatment of choice [10,11].

This report is limited by the absence of endocrine assays and immunohistochemistry, with the diagnosis resting on the clinical picture, ultrasonographic findings, and characteristic histology, and by the fact that postoperative sexual function could not be assessed.

## Conclusions

Mucous cysts arising within the clitoris are exceptionally rare, to our knowledge, with only one previous case reported; the present case represents the second such documented occurrence. Because these lesions can mimic other clitoral and vulvar masses both clinically and on gross inspection, they warrant inclusion in the differential diagnosis of a clitoral swelling, and histopathological examination is required for definitive diagnosis. As demonstrated here, the cyst tends to compress rather than replace the clitoral glans; complete surgical excision with preservation of the glans and its neurovascular supply therefore achieves curative resection with satisfactory cosmetic and functional outcomes, whereas simple aspiration is unlikely to be definitive.
